# Identifying strengths and weaknesses of the integration of biomedical and herbal medicine units in Ghana using the WHO Health Systems Framework: a qualitative study

**DOI:** 10.1186/s12906-018-2334-2

**Published:** 2018-10-22

**Authors:** Bernard Appiah, Isaac Kingsley Amponsah, Anubhuti Poudyal, Merlin Lincoln Kwao Mensah

**Affiliations:** 1 Research Program on Public and International Engagement for Health, Department of Environmental and Occupational Health, School of Public Health, Texas A&M University, College Station, TX 77843-1266 USA; 2 Department of Pharmacognosy, Faculty of Pharmacy and Pharmaceutical Sciences, Kwame Nkrumah University of Science and Technology, Kumasi, Ghana; 3 Department of Health Promotion and Community Health Sciences, School of Public Health, Texas A&M University, College Station, TX 77843-1266 USA; 4 Department of Herbal Medicine, Faculty of Pharmacy and Pharmaceutical Sciences, Kwame Nkrumah University of Science and Technology, Kumasi, Ghana

**Keywords:** Herbal medicine, Research, Biomedicine, Integration, Qualitative research, Ghana

## Abstract

**Background:**

The use of herbal medicines in developing countries has been increasing over the years. In Ghana, since 2011, the government has been piloting the integration of herbal medicine in 17 public hospitals. However, the strengths and the weaknesses of the integration have not been fully explored. The current study sought to examine the strengths and weaknesses of the integration using the WHO health systems framework.

**Methods:**

This study used qualitative, exploratory study design involving interviews of 25 key informants. The respondents had experience in conducting herbal medicine research. Two key informants were medical herbalists practising in hospitals piloting the integration in Ghana. We used Framework analysis to identify the perspectives of key informants in regards to the integration.

**Results:**

Key informants mostly support the integration although some noted that the government needs to support scale-up in other public hospitals. Among the strengths cited were the employment of medical herbalists, utilization of traditional knowledge, research opportunities, and efficient service delivery by restricting the prescription and use of fake herbal medicine. The weaknesses were the lack of government policies on implementing the integration, financial challenges because the National Health Insurance Scheme does not cover herbal medicine, poor advocacy and research opportunities, and lack of training of conventional health practitioners in herbal medicine.

**Conclusions:**

Researchers view the integration of the two healthcare systems–biomedicine, and herbal medicine– positively but it has challenges that need to be addressed. The integration could offer more opportunities for researching into herbal medicine. More training for conventional health professionals in herbal medicine could increase the chances of better coordination between the two units. Additionally, strong advocacy and publicity is needed to educate more people on the integration and the utilization of the services.

**Electronic supplementary material:**

The online version of this article (10.1186/s12906-018-2334-2) contains supplementary material, which is available to authorized users.

## Background

Herbal medicine use is common worldwide. For example, the World Health Organization estimates that 80% of the population worldwide use herbal medicines [1]. Herbal medicine is a subset of traditional medicine. While traditional medicine includes practices such as spiritism and soothsaying to address healthcare challenges, herbal medicine focuses on use of herbs or herbal remedies to control diseases [2]. Given the increasing use of herbal medicine, some countries such as China have integrated herbal medicine and biomedical medicine within their healthcare system [2]. The integration of traditional medicine into the biomedical healthcare system has challenges such as perceived efficacy of herbal remedies and lack of coverage of herbal medicines by health insurance [3]. There could be economic and prestige competition between the two health care systems [4, 5]. Additionally, some Western-trained healthcare practitioners may view herbal remedies as being harmful to patients [5].

Ghana’s healthcare system is said to be pluralistic as it combines both traditional and biomedical systems [6, 7]. People’s perceptions of the potential causes of a disease may let them use the traditional or the biomedical systems [7].

The traditional medical system in Ghana includes the use of herbalists, priests and traditional healers [6, 7]. The modern biomedical healthcare system is operated or financed by the government although the private sector such as religious missions also operate healthcare services. Moreover, the military or large firms provide their employees quasi-government-operated health services. The Ministry of Health manages the traditional and biomedical systems. Individuals who seek care from biomedical systems pay for those services if they are not covered by the National Health Insurance Service (NHIS) [8]. The NHIS does not cover healthcare provided by the traditional systems.

In Ghana, the importance of herbal medicine in healthcare delivery has led to at least five major interventions since 2001. First, the Kwame Nkrumah University of Science and Technology started a new bachelor’s programme in herbal medicine in 2001, and by 2012 had trained 112 medical herbalists [9]. Second, Ghana’s Ministry of Health established a Traditional and Alternative Medicine Directorate in April 2001 [10]. Third, in part through the efforts of the Directorate, recommendations were made for Ghanaian public hospitals to prescribe some herbal medicines [10]. Fourth, in 2005, the country launched a new policy on the practice of alternative medicine [11]. Finally, in 2011, the government began a pilot project in 17 public hospitals for integrating herbal medicine into biomedicine [9]. However, more than six years into the pilot project, there appears to be limited research that explores the integration. A recent case study of one of the hospitals enumerated the history of the integration, but did not discuss opportunities or challenges of the integration [12]. There have been several studies in Ghana about use of herbal medicines from the perspectives of patients [13–18] but research studies on the pilot interventions are scarce.

Therefore, this study sought to identify the strengths and weaknesses of the 2011 government-initiated integration of herbal medicine into the existing biomedical practice in Ghana using the WHO Health Systems Framework.

The WHO Health Systems Framework analyzes the healthcare system based on six system building blocks – leadership/governance, healthcare financing, health workforce, medical products & technologies, information & research, and service delivery [19]. Under leadership and governance, the existence of strategic policy frameworks, along with appropriate regulations in place within a health system is assessed. Healthcare financing identifies adequate funds that are available to ensure people can access the necessary services. This includes financial protection and support to those who cannot afford healthcare. Health workforce consists of staff members in adequate number. Additionally, it looks at the efficiency, responsiveness, fairness and competence of the workforce. Medical products and technology comprise equitable access to essential medical products, with assurances of quality, safety, and cost-effectiveness. Information and research ensures an efficient production, analysis and dissemination of information related to health systems. Service delivery assures effective and safe health services for those who need them [19]. The WHO Health Systems Framework has been used to investigate universal health coverage [20], vertical programs [21], and integration of mental health services in primary care [22]. However, to the best of our knowledge, it has not been used to assess integration of herbal medicine units with biomedical units.

In order to identify the strengths and weaknesses of the integration, we sought the views of researchers in fields such as social science, public health and herbal medicine. Additionally, we interviewed medical herbalists who are currently working in two of the piloted hospitals that are running herbal medicine and biomedical units. This study was part of a larger project that aimed to create a socio-behavioral research strategy for conducting community-based studies on herbal medicine in Ghana.

## Methods

### Ethical approval

The research ethics application for conducting this study was reviewed and approved by the Institutional Review Boards of the Kwame Nkrumah University of Science and Technology in Ghana and the Texas A&M University in the United States. All participants provided written consents to participate in the study.

### Study design

We used a qualitative study design in part because of the paucity of information on the subject [23]. We used key informant interviews because such an approach made it possible for meeting interviewees at their most convenient times. Interviews could facilitate generation of information by helping the interviewee recall facts and express recollections [24].

### Study population

We purposively selected interviewees with interest in conducting herbal medicine research. We identified four study sites, namely, schools of the Kwame Nkrumah University of Science and Technology (KNUST), Kumasi; Centre for Plant Medicine Research (CPMR), Mampong-Akuapem and two hospitals that have piloted integration of herbal medicine and biomedicine. A total of 25 participants took part in the study. Among them, 18 participants were from the Faculty of Pharmacy and Pharmaceutical Sciences, two were from the School of Public Health, one was from the College of Science and one was from the College of Arts and Social Sciences, all under KNUST. One participant was interviewed from CPMR and one each from two of the piloted hospitals.

### Data collection

Telephone calls to key herbal medicine researchers resulted in them agreeing to participate. We used snowball sampling to identify further researchers. The process of interviewing occurred until “saturation” point was reached [25]. All the interviews lasted from 19 to 49 min, were audiorecorded and transcribed. BA conducted the interviews in English because all interviewees were literate in English. BA is a pharmacist with extensive experience in conducting qualitative research. The interviews took place in August 2016. With the exception of one of the medical herbalists who was interviewed at his workplace, an herbal medicine unit at a hospital, all the other participants— including the one from CPMR— were interviewed at the KNUST.

### Data analysis

We used Framework analysis [26] to identify the perspectives of the interviewees in regards to the integration. Framework analysis involves five steps – familiarization; identifying a thematic framework; indexing, charting; and mapping and interpretation. Under familiarization, the researchers read the transcripts and took notes on the important ideas. A thematic framework was then generated by two of the researchers (BA and AP) based on the discussion and notes taken during the familiarization phase. BA has training in advanced qualitative research methods; AP is a graduate student in health promotion and community health sciences who has experience in analyzing qualitative data. To identify the broad themes, we initially focused on the interview questions [27]. Later, key themes from the transcripts emerged slightly different from pre-determined codes [28, 29]. After identifying the thematic framework, indexing was done in which certain portions of the transcripts were marked as belonging to certain themes. Then, charting involved arranging the pieces of information in charts according to the themes with the aid of Microsoft Excel. Three researchers (BA, AP and IKA) identified quotes relevant to the themes, discussed them and where discrepancy occurred, resolved them. All the members of the research team approved the quotes and the themes. Finally, mapping and interpretation was done in which the charted information was arranged to identify the nature of the integration, opportunities and challenges of the integration process and potential hospital- and community- based studies that can provide evidence to effectively guide policy on the integration in Ghana. The interview questions can be found in the Additional file 1.

## Results

Of the 25 participants interviewed, two were medical herbalists with experience in conducting research; one was a social scientist who has taught African traditional medicine course; two were public health researchers who had conducted studies into herbal medicine; one was a biochemist with experience in exploring chemical properties of herbs; and one was a traditional herbalist who also works as a researcher at the KNUST. The rest were herbal medicine researchers at the Faculty of Pharmacy and Pharmacy and Pharmaceutical Sciences at the KNUST.

The study assesses the strengths and weakness of integrating biomedical and herbal medicine in Ghana using the WHO health systems framework.

### Leadership/governance

Most respondents considered the integration as an important step towards formalizing herbal medicine in Ghana with participants calling it “a laudable idea”, “a bold attempt”, and “a right step” for the country. However, respondents mentioned the lack of administrative procedures and clear explanation of the government policy on integration, which hindered implementation.

*“[There is a need to] set up a good administrative procedure.”* [Interview 2]

A weakness stated by many was the delay in the integration process from the government side, with little to no efforts made at initiating integration in hospitals at the peripheral level. Even though the need for herbal medicines was higher among the people in rural areas compared to those in urban areas, the integration was mostly done in urban hospitals.

*“The pace at which the government is moving is very slow.”* [Interview 8]

*“The government should speed up the Herbal Units and even let the Teaching, Municipal and the District hospitals have them.”* [Interview 7]

*“I think it should rather be in the District hospitals instead of the bigger ones [hospitals] in Kumasi and in Accra because it is the local people who believe so much in the herbal medicines.”* [Interview 25]

### Health care financing

One of the biggest challenges of the integration as stated by the respondents is that the National Health Insurance Scheme does not cover the few selected herbal medicines used by the herbal medicine units. Patients who opt for herbal treatment have to pay out of pocket compared with those who sought treatment from orthodox doctors.

*“Since the hospital management has not accepted the specific roles these [herbal units] are to play, [herbal products] are not [available] under the National Health Insurance Scheme (NHIS), and patients pay fully out of their pocket for medicines that are from the herbal units or departments.”* [Interview 11]

*“We should get the herbal medicines listed on the National Health Insurance Scheme.”* [Interview 13]

### Health workforce

One of the strengths of the integration was the potential for new job opportunities for medical herbalists, who graduated with formal training in herbal medicine. With an herbal unit in place, respondents were optimistic about the increase in job opportunities. One respondent said, “*It has offered employment for the graduate*” [Interview 3]. However, at present, few conventional health professionals (CHPs) have training in herbal medicine, as pointed out by a few respondents.

*“We have to start from our schools, the Medical schools. The [conventional health practitioners] should know what the herbs can do, they should understand what herbalism is or traditional medicine treatment or herbal treatment*…*Re-structure the Medical school curriculum, and also those of the paramedics, including the nurses.”* [Interview 10]

When discussing the current staff members in the herbal and biomedical units, it was suggested that healthcare professionals in the biomedical units did not trust the herbal units or the integration process. Thus, patients were not referred to herbal units as often as the other way around.

*“In some of the hospitals the [herbal] unit is not accepted. They [administrators and doctors] feel they [herbal units] don’t have anything to offer.”* [Interview 10]

Respondents agreed that there was a need for medical herbalists along with CHPs with training in herbal medicine to enable the integration’s scale-up.

*“[You need to] train people [with] herbal medicine background to [become] medical doctors.”* [Interview 4]

*“Each of them [medical doctors and nurses should be given courses on] traditional medicine, for them to appreciate traditional medicine.”* [Interview 3]

### Medical products, technologies

A big boost to the integration is the abundance of traditional knowledge and herbal medicine use in Ghana. However, the participants mentioned difficulty in registering such herbal medicines because of limited funding for efficacy studies and safety trials. With little evidence on efficacy of the herbal medicine, there were limited registered herbal products. The difficulty in registration has also led to poor accessibility of herbal medicine.

*“They [Food and Drug Authority] will not register anything [without] subacute or chronic toxicity [tests] and if they continue doing this, it will sabotage the herbal industry.”* [Interview 5]

Some participants felt that the lack of systematic approach to obtaining herbs, could pose as a potential threat that can lead to extinction of plants used for producing herbal medicines.

*“Sit down with all stakeholders, bring ideas and see how we can ensure the continuous supply of herbal raw materials to support the programme.”* [Interview 19]

*“I don’t think there has been a serious consideration given to [conservation] of raw material sources [medicinal plants].”* [Interview 19]

### Information and research

A strength mentioned about the integration was the potential to conduct research in the hospitals where biomedical and herbal medicine have been integrated. Because there are limited studies on herbal medicine use, especially in combination with biomedical medicine, study sites where both services were provided were considered important.

*“Most of the [herbal] drugs that we use seem to be based on anecdotal evidence of efficacy.”* [Interview 7]

*“[Integration] gives room for research, why people are using this in combination with other products, what are the results, the effects so it’s a good idea.”* [Interview 12]

**Fig. 1:**
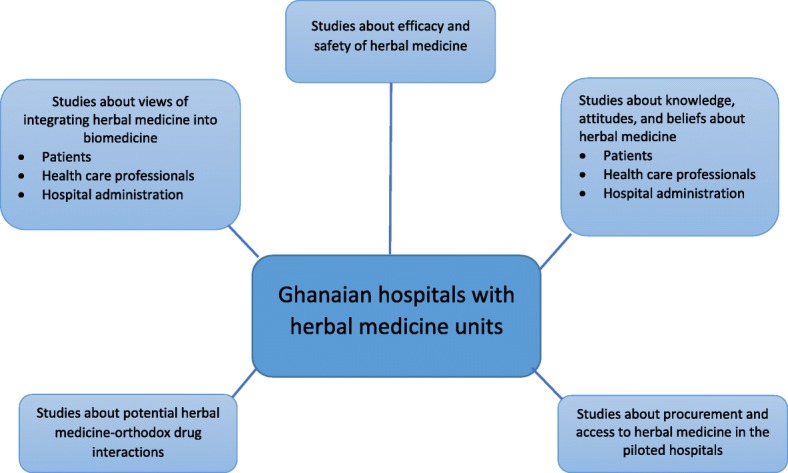
Types of studies to be conducted in Ghanaian hospitals piloting herbal medicine biomedicine integration

Respondents cited five main research topics for consideration: studies focusing on the views of integrating herbal medicine and biomedicine, studies about efficacy and safety of herbal medicines, studies about knowledge, attitudes and beliefs about herbal medicine, studies about potential interactions involving herbal medicine and orthodox medicine and studies about procurement and access to herbal medicines (Fig. 1). For example, some participants noted that the existence of herbal departments in the pilot hospitals offers unique opportunities for conducting studies that focus on herbal medicine from the perspectives of patients and health professionals, and efficacy and safety trials.

*“Let’s [conduct ethnobotanical surveys] to seek some ideas from herbalists so that they don’t die with the knowledge.”* [Interview 18]

One challenge noted by the respondents was the lack of publicity to educate the public on the integration and the services they can access at the hospitals.

“*Publicity is very low.”* [Interview 25]

*“Platforms [are needed] for us to let whatever findings we’ve gotten in our various clinics to be communicated to the public.”* [Interview 1]

*“I think adverts on radio and then maybe having flyers and posters [on the integration] will do.”* [Interview 2]

### Service delivery

It was suggested that the integration could facilitate effective service delivery by negating the potential increase in the number of fake doctors, particularly those prescribing counterfeit herbal medicine. Presently, people reach out to traditional healers in an informal setting. A regulated integration could help people in accessing reliable herbal medicine services. A respondent said, *“It is going to help streamline the use of herbal medicines in treating diseases and it will also eliminate the quacks from the system”* [Interview 8].

With the integration, one interviewee reported that he expected more coordination in service delivery. He said, “*what we would have wanted to see is when somebody gets to these hospitals, the options are explained to him and the patient decides to go and when he’s gone, the practitioners should be able to consult each other when they are in difficulty”* [Interview 9].

However, some respondents were concerned that the referral of the patients from herbal medicine unit to biomedical unit was much higher than the other way around. The latter was only done when orthodox medicine did not work.

*“They only go there [herbal unit] when … the orthodox treatment is not working.”* [Interview 2]

Respondents mentioned a sense of mistrust between the two units. Rather than working in unison, these two units were working independently, with a sense that the other unit is unwanted in the setting.

*“Assuming [an] orthodox medicine was applied and it didn’t work and [the] herbal one works, don’t you think people will lose confidence in scientific medicine? And if it happens that way it’s going to cause serious problems.”* [Interview 18]

*“[Orthodox] medics didn’t want any such thing [integration] to happen.”* [Interview 13]

A respondent said that there is a need to document referrals from herbal unit to biomedical unit and vice-versa to get a clear picture of the kind of patients treated in each unit.

*“What we refer to the orthodox [doctors] and what they refer here [herbal medicine unit] need to be recorded.”* [Interview 1]

Another challenge in service delivery was that the residents from rural villages might not use the herbal medicine services in urban areas if they can get herbal medicines from traditional healers in their own communities.

*“The village resident will not come from the village to the hospital to seek herbal treatment.”* [Interview 20]

## Discussion

Ghana’s inclusion of herbal medicine units in 17 piloted public hospitals demonstrates the resolve of the country in prioritizing herbal medicine in the country’s healthcare system. The current study offers insights into strengths and weaknesses of the integration as assessed using the WHO health systems framework. This study could help improve such integration project in Ghana, and other African countries that may be interested in similar initiatives.

An important finding of the study is the role of the government in facilitating integration after the policy has been made. Following the 2005 policy on the practice of integrated medicine, a previous study in Ghana reported an absence of protocol that outlined the definition, process and goals of integration, which caused disagreement on how and when services were considered integrated [12]. The current study also identified insufficient approaches from the government in explaining administrative procedures. The WHO calls for deliberate policy decisions to ensure that traditional and Western medicine complement each other following the integration [30]. Therefore, clear goals, along with procedures in the most efficient utilization of integration, can direct the healthcare professionals to maximize this opportunity.

Furthermore, the intervention was not designed to fit into the key national health policy in Ghana: the National Health Insurance Scheme. In Ghana, the NHIS had led to increased utilization of health services [31–33]. Given that medicines that are available on the NHIS list are not to be paid for by patients, the lack of herbal medicines on the list poses a serious threat to the integration. Any effort to improve the pilot project may need to consider at least a few herbal medicines with proven safety and efficacy profiles for diseases of common occurrence such as malaria to be included on the list. Many countries have included traditional medicine into their healthcare system. For instance, in China traditional medicines are covered by public and private insurance and are practiced along every level of health care service. Similarly, in Republic of Korea and Viet Nam, traditional medicine practitioners can practice in both public and private hospitals and the insurance covers the services. An important step in covering herbal medicine in the NHIS can be determining the medicines or services that are covered by the NHIS. For instance, in Switzerland following the integration, the compulsory health insurance program covered five complementary therapies if the physician prescribing the medication was certified in complementary and alternative medicine [2].

Another potential view worthy of discussion is the role of advocacy: it appears that lack of publicity for the pilot project has limited public understanding of the integration and its implementation. Ghana’s policy on alternative medicine recommends training of media professionals to help educate the public on the efficacy and dangers of herbal medicine [11], but perhaps the media may need to be actively involved in promoting the integration.

Furthermore, all institutions that train healthcare professionals in the country must consider introducing herbal medicine as a course for their students as recommended by the WHO [34]. It is noteworthy that the West African Health Organization has developed a curriculum on traditional medicine for the sub-region’s medical schools. While Nigeria appears to have embraced it [35], Ghana is yet to introduce it at the undergraduate level. Lack of training on herbal medicine can hinder the integration by limiting the number of medical herbalists or CHPs with herbal medicine training. Understanding traditional medicine is imperative for CHPs because it helps them understand the health services accessed by the patients and provide informed suggestions on health and health-related issues [36].

An important finding of this study is the research opportunity that the integration offers. The types of studies enumerated by participants suggest a strong need for social science research methodology training among current and future herbal medicine researchers. For example, seeking the knowledge, attitudes and beliefs about herbal medicine among patients, healthcare professionals and hospital administration may require qualitative and quantitative research approaches.

The piloted hospitals also offer natural environment for conducting efficacy and safety studies on herbal medicine for both communicable and non-communicable diseases. The cost of medicines is a serious impediment to accessing healthcare. If authorities pay attention to determining what herbal medicines work best and are safe to use for medical problems such as diabetes, malaria and hypertension, for instance, Ghana and other African countries could help increase access to safe and efficacious herbal medicines for such medical problems.

Moreover, for the integration to be successful there is a need to engage many key actors such as patients, health professionals, policymakers, farmers and media. One of the major findings of the current study is the fact that some medicinal plants are becoming extinct. This finding suggests a need for making serious efforts towards conserving plants used for herbal medicine [34]. Farmers and agricultural scientists may therefore need to be engaged to help find ways of conserving herbs that serve as raw materials for herbal medicines. In other words, herbal medicine researchers, social scientists and agricultural scientists may need to work together to help address key research issues that could help improve the integration. In addition, the involvement of the mass media in creating awareness of the integration should not be downplayed. There is a need to engage the media and to train them to adequately report on challenges and solutions of the integration. A holistic review of the integration should be conducted to help facilitate its potential scale-up.

A major limitation of this study is that most participants are researchers based at the Kwame Nkrumah University of Science and Technology (KNUST), thus limiting other perspectives of researchers in other institutions with interest in herbal medicine, and those of policymakers. However, given that KNUST is the only institution in Ghana that offers a bachelor’s degree in herbal medicine at present, involving herbal researchers in this institution might have enriched this study. Moreover, because of the difficulty of organizing researchers together, focus group discussions were not conducted to help complement the key informant interviews. Finally, since three of the authors of this paper are researchers familiar with the integration, their experiences and assumptions might influence the interviewees’ responses on the integration. Future research should consider other key actors such as policymakers and patients, and use of qualitative and quantitative methods to explore the subject further.

## Conclusion

This study shows that researchers view Ghana’s pilot intervention involving the introduction of herbal medicine into public hospitals generally positively although some indicate that the government needs to do more to accelerate its scale-up across the country. There appears to be challenges that should be addressed to facilitate the success of such an innovative intervention. This study identifies potential research opportunities and makes recommendations such as a need for a revised policy on the integration; a need for the country’s National Health Insurance Authority to consider having key herbal medicines on the National Health Insurance Scheme to facilitate patronage of the herbal units in such hospitals, and the need to train conventional health practitioners and medical students in herbal medicine.

## Additional file

Additional file 1: Interview guide used for the qualitative study. (DOCX 14 kb)

## Abbreviations

CHPs: Conventional Health professionals
CPMR: Centre for Plant Medicine Research
KNUST: Kwame Nkrumah University of Science and Technology (KNUST)
NHIA: National Health Insurance Authority
NHIS: National Health Insurance Scheme
WHO: World Health Organisation
