# Supplementary material for: Identifying strengths and weaknesses of the integration of biomedical and herbal medicine units in Ghana using the WHO Health Systems Framework: a qualitative study
Source: BMC Complement Altern Med. 2018 Oct 22;18:286. doi: 10.1186/s12906-018-2334-2 (PMC6196414; doi:10.1186/s12906-018-2334-2)
Supplement: Supplementary file 1 — Interview guide used for the qualitative study. (DOCX 14 kb) [file 12906_2018_2334_MOESM1_ESM.docx]

**Supplemental Material to “Identifying strengths and weaknesses of the integration of biomedical and herbal medicine units in Ghana using the WHO Health Systems Framework: a qualitative study”**

**Bernard Appiah,* Isaac Kingsley Amponsah^2^, Anubhuti Poudyal^3^, Merlin Lincoln Kwao Mensah^4^**

1. Research Program on Public and International Engagement for Health, Department of Environmental and Occupational Health, School of Public Health, Texas A& M University, College Station, TX, USA: [appiah@sph.tamhsc.edu](mailto:appiah@sph.tamhsc.edu)
2. Department of Pharmacognosy, Faculty of Pharmacy and Pharmaceutical Sciences, Kwame Nkrumah University of Science and Technology, Kumasi, Ghana: [akila.amponsah@gmail.com](mailto:akila.amponsah@gmail.com)
3. Department of Health Promotion and Community Health Sciences, School of Public Health, Texas A& M University, College Station, TX, USA: [anubhuti.p3@gmail.com](mailto:anubhuti.p3@gmail.com)
4. Department of Herbal Medicine, Faculty of Pharmacy and Pharmaceutical Sciences, Kwame Nkrumah University of Science and Technology, Kumasi, Ghana: mlkmensah@yahoo.com

*Corresponding author

Name: Dr. Bernard Appiah

Postal address: Texas A&M University School of Public Health, 212 Adriance Lab Rd 1266 TAMU College Station, TX 77843-1266

Email: appiah@sph.tamhsc.edu

Telephone number: 1.979.436.9456

**Additional file 1.**

**Interview guide used for the qualitative study**

In Ghana, a few public hospitals are piloting integrated herbal medicine use by having herbal clinics in such facilities.

1. What are your views on the implementation of the programme?
2. What are the challenges of the implementation of this pilot programme, if any?
3. What are the successes of the implementation of this pilot programme, if any?
4. What are the solutions to addressing the challenges, if any?
5. What research topics can be conducted in hospitals piloting the programme?
6. What research topics can be conducted in communities with hospitals piloting the programme?
7. Any other information on the pilot programme?
